# Adjuvant Chemotherapy for Patients with Stage III Colon Cancer: Results from a CDC-NPCR Patterns of Care Study

**DOI:** 10.4137/cmo.s2316

**Published:** 2009-11-24

**Authors:** Rosemary D. Cress, Susan A. Sabatino, Xiao-Cheng Wu, Maria J. Schymura, Randi Rycroft, Erik Stuckart, John Fulton, Tiefu Shen

**Affiliations:** 1 California Cancer Registry, Public Health Institute, Department of Public Health Sciences, Division of Epidemiology, University of California Davis, Sacramento, CA, USA.; 2 Division of Cancer Prevention and Control, Centers for Disease Control and Prevention, Atlanta, GA, USA.; 3 Louisiana State University Health Sciences Center, New Orleans, Louisiana, USA.; 4 New York State Cancer Registry, New York State Department of Health, Albany, NY, USA.; 5 Colorado Central Cancer Registry, Colorado Dept. of Public Health and Environment, Denver, CO, USA.; 6 Dept. of Health Informatics, Medical College of Georgia, EB-1010, Augusta, GA, USA.; 7 Rhode Island Cancer Registry, Rhode Island Department of Health, Providence, RI, USA.; 8 Division of Epidemiologic Studies, Illinois Department of Public Health, Springfield, IL, USA.

**Keywords:** colorectal cancer, adjuvant chemotherapy, patterns of care

## Abstract

**Objective:**

To evaluate adjuvant chemotherapy use for Stage III colon cancer.

**Methods:**

This analysis included 973 patients with surgically treated stage III colon cancer. Socioeconomic information from the 2000 census was linked to patients’ residential census tracts. Vital status through 12/31/02 was obtained from medical records and linkage to state vital statistics files and the National Death Index.

**Results:**

Adjuvant chemotherapy was received by 67%. Treatment varied by state of residence, with Colorado, Rhode Island and New York residents more likely to receive chemotherapy than Louisiana residents. Older age, increasing comorbidities, divorced/widowed marital status, and residence in lower education areas or non-working class neighborhoods were associated with lower chemotherapy use. Survival varied by state but after adjustment for sex, sociodemographic and health factors, was significantly higher only for California and Rhode Island. Older age and lower educational attainment were associated with lower survival. Chemotherapy was protective for all comorbidity groups.

**Conclusion:**

Although adjuvant chemotherapy for Stage III colon cancer improves survival, some patients did not receive standard of care, demonstrating the need for cancer treatment surveillance. Interstate differences likely resulted from differences in local practice patterns, acceptance of treatment, and access.

## Introduction

Colon cancer, exclusive of rectal cancer, killed approximately 45,000 people in the U.S. in 2004^1^ making it the second most common cause of cancer death among men and women combined. Although screening and early detection are the best way to prevent death from this disease, adjuvant chemotherapy (chemotherapy given after surgical management) has been shown to improve survival. A National Institutes of Health consensus conference in 1990 addressed which colorectal cancer patients were at risk for recurrence after surgery, and whether adjuvant chemotherapies were effective. Recommendations varied by stage, with a recommendation for chemotherapy after surgery for eligible patients with Stage III colon cancer without contraindications.^2^ However, prior studies have suggested that many patients do not receive stage-appropriate treatment.^3^

The Centers for Disease Control and Prevention’s National Program of Cancer Registries (CDC-NPCR) Patterns of Care (POC) study was organized as the result of two reports from the Institute of Medicine (IOM).^4^,^5^ The National Cancer Policy Board of the IOM concluded that some individuals may not be receiving effective treatment for cancer, and recommended that data systems such as the NPCR be used to conduct surveillance of cancer treatment in the United States. Cancer registries routinely collect information on first course of treatment. However data on chemotherapy may be missing, particularly if administered in outpatient facilities. CDC and state investigators collaborated to assess both quality of treatment data in state cancer registries, and receipt of appropriate treatment by cancer patients in those states. The POC study enabled a reabstraction of registry data on the characteristics and treatment of localized female breast cancer, regional colon cancer, and localized prostate cancer in the United States.^6^ For the first time, the POC study allowed a large-scale evaluation of data among several state population-based cancer registries supported by CDC-NPCR. The objective of this study was to evaluate the use of adjuvant chemotherapy for surgically treated patients diagnosed with Stage III colon cancer resident in seven states covered by the CDC-NPCR program as part of the larger study of patterns of care.

## Methods

Administered by CDC, NPCR collects data on cancer incidence through central cancer registries in 45 states, the District of Columbia, Puerto Rico, the Republic of Palau and the Virgin Islands. In this study, staff from seven population-based state cancer registries (California, Colorado, Illinois, Louisiana, New York, South Carolina, Rhode Island) collected supplemental treatment data for a random sample of cancer patients diagnosed in 1997.^7^ Patients included in the current analysis were those diagnosed with a first primary Stage III colon cancer (ICD-O site codes C18.0–18.9), defined as American Joint Commission on Cancer pathology stage at diagnosis of 3, 3A, 3B, 3C and Summary Stage 3 or 4 according to SEER Summary Stage 1977.^8^ Only patients with adenocarcinoma or adenosquamous carcinoma who received surgical treatment for their colon cancer were included. Patients diagnosed with a subsequent cancer within four months of their colon cancer diagnosis, and patients whose date of death or last contact was within 30 days of surgery, were excluded.

Trained abstractors gathered information on treatment and other factors from patient medical records, including hospital charts and records from physician offices, ambulatory surgery and radiation therapy facilities. ICD-9-CM codes recorded in medical records were extracted to assess the presence of comorbid illness, which would influence receipt of treatment. Comorbid illness burden was assessed using the Charlson comorbidity index, which yields a score based on the number of conditions present, and the weight for that condition. Codes for the 17 conditions that make up the Charlson index were consolidated into a summary Charlson measure.^9^ Race and ethnicity information was combined to create four groups: Hispanic, non-Hispanic white, non-Hispanic black, and non-Hispanic other (including Asian/Pacific Islander) or unknown. Patients were categorized as non-Hispanic unless there was evidence to the contrary.

Each patient’s address at the time of diagnosis was geocoded to the appropriate census tract within the state of residence. Information on socioeconomic status (poverty, education and occupation) and urban vs. rural residence from the 2000 census was assigned to each census tract and linked to the patient’s census tract of residence. Patients residing in census tracts where fewer than 25% of adults had a high school education were classified as of lower education attainment; those residing in census tracts where at least 66% of adults were in a working class occupation were classified as working class; and those residing in census tracts where at least 20% of residents lived below the 2000 Federal Poverty Level were classified as in poverty.^10^

Insurance status was determined from medical records. Private insurance included those with either private insurance or Medicare with a supplement. Public insurance included those with Medicare, Medicaid or welfare, or other federally funded health insurance. Vital status as of 12/31/02 was obtained from medical records and physician offices, and was enhanced by linking to state vital statistics files and to the National Death Index.

### Analysis

We examined the proportions of patients receiving adjuvant chemotherapy by sociodemographic and health factors for the entire sample and after stratifying by sex. Fifty-eight patients who were missing information about receipt of chemotherapy were excluded from the analysis of treatment receipt. In addition, we used multivariable logistic regression modeling to examine the crude and adjusted odds ratios with 95% confidence intervals of receiving adjuvant chemotherapy. Odds ratios were adjusted for age at diagnosis, sex, race/ethnicity, marital status, registry, health insurance, urban-rural residence, poverty, education, working class status, and Charlson comorbidity score. Model goodness of fit was tested with the Hosmer-Lemeshow test. Reference categories were generally chosen to be those with the greatest numbers, except where selected to facilitate comparisons with other studies.

Finally, we examined survival from diagnosis through December 31, 2002. First, we estimated the proportion of patients surviving 5 years by receipt of chemotherapy, sex, sociodemographic and health factors. In addition, we used Cox proportional hazards models to estimate crude and adjusted hazards ratios with 95% confidence intervals, adjusting for all factors. We considered death due to any cause as an event. Because patients who received chemotherapy survived at least long enough to receive treatment, a time-varying covariate for receipt of adjuvant chemotherapy was included in the models to reduce potential bias. Time-varying covariates also were used to test the proportional hazards assumption, which was violated for age at diagnosis. Therefore a time-varying covariate for age was kept in the model. We also examined an interaction between receiving adjuvant chemotherapy and comorbidity status. For dates, missing information about day of the month (n = 27) was coded to 15 for February and to 16 for all other months. Missing information about month (n = 1) was left as missing. SAS statistical software (version 9.1, SAS Institute, Cary, NC) was used in all analyses. P values < 0.05 were considered statistically significant.

## Results

A total of 1066 patients with Stage III colon cancer were identified. Patients with histologic types other than adenocarcinoma or adenosquamous carcinoma were excluded (n = 5) as well as one patient who did not receive surgical treatment, 20 patients diagnosed with a subsequent cancer within four months of their colon cancer diagnosis, and 67 patients whose date of death or last contact was within 30 days of surgery. Our final sample included 973 patients. Characteristics of the study population are shown in Table 1. Slightly more than half (53%) were female and the majority of patients were non-Hispanic white (76%), aged 65 or older (61%), and lived in urban areas (67%). Most had private insurance (69%) and the majority had no indication of comorbid illness in the medical record (75%).

Patients were classified as being treated if information in the medical record indicated they had received any chemotherapy. Table 2 shows the unadjusted percent distributions of receipt of chemotherapy by sociodemographic and health factors. A total of 67% of eligible patients had a record of receipt of adjuvant chemotherapy. Patients under age 50 had the highest proportion of treatment (86%) and this declined to 41% among patients aged 75 and older. Differences in treatment by race group were non-significant; receipt of adjuvant chemotherapy was slightly higher among males than females in all race groups except non-Hispanic blacks where women had a higher rate of treatment. The proportion of patients treated was higher for married patients. The proportion of patients who received adjuvant chemotherapy varied by their state of residence, from 59% to 73%, although differences were not statistically significant. Patients with public insurance had the lowest likelihood of treatment, while those with no known health insurance had a higher proportion of treatment, although only a small number of patients were included in this category. Patients residing in rural communities had a non-significantly higher likelihood of treatment, as did patients residing in census tracts where a higher proportion of residents were educated. Patients with two or more comorbid illnesses recorded in their medical records were less likely to have received adjuvant treatment than those with no comorbid illness.

Crude and adjusted odds ratios for receipt of adjuvant chemotherapy in this population are shown in Table 3. After adjustment for other variables, state of residence was the strongest predictor of receipt of adjuvant treatment, and patients residing in three states (Colorado, Rhode Island, New York) were about twice as likely as those in the reference state (Louisiana) to have received treatment. Age also was a powerful predictor of receipt of adjuvant treatment, and patients aged 75 and older were only 24% as likely to have received this treatment compared to those aged 65 to 74. Patients with two or more comorbid illnesses were about one-third as likely as those with no comorbidity to have received treatment. Divorced or widowed patients were about half as likely as married patients to have received treatment. Patients who lived in census tracts with lower educational attainment were less likely to have received adjuvant therapy, however those living in working class neighborhoods were more likely to have received treatment after adjusting for other factors, and census tract level of poverty did not affect probability of treatment. Race/ethnicity, gender, urban/rural residence, and health insurance did not have a statistically significant effect on treatment after adjustment for other variables.

Five year survival rates and crude and adjusted hazard ratios are shown in Table 4. Eighty-nine patients were missing information for the time-varying covariate or receipt of adjuvant chemotherapy and were excluded from all survival analyses. One additional individual was missing information about date of diagnosis, and therefore survival time could not be calculated. Therefore, data for these analyses were available from 883 patients. Patients aged 75 and older had a substantially higher risk of death from any cause in five years, and men had a higher risk of dying than did women (p = 0.06). Formerly married patients had a higher risk of death but this was not statistically significant after adjustment for age and other factors. Five year survival rates varied by state of residence, from 45% to 63%, but the hazard ratio was statistically significantly lower (survival significantly higher) only for California and Rhode Island after adjustment for other factors. Patients with public insurance or without insurance had a higher risk of death than those with private insurance, but again this result was not statistically significant after adjustment. Patients with lower educational attainment also had a higher risk of death than those with higher educational attainment. Crude hazard ratios show that patients with any comorbid condition were more likely to die than those with no comorbid illnesses, and those with two or more comorbid illnesses were approximately twice as likely to die. Unadjusted findings indicate that almost twice as many patients who received adjuvant chemotherapy survived five years (61% versus 33%). In multivariate analysis, a statistically significant interaction was found between receipt of adjuvant therapy and comorbidity status (p < 0.025). Receiving chemotherapy was protective in all comorbidity groups, with the strongest effect among patients with a Charlson score of one. Among those who received chemotherapy, increasing comorbid illness burden was not associated with significantly lower survival. However among patients who did not receive chemotherapy, patients with comorbid illnesses had lower survival than patients with no comorbidities. No statistically significant differences in survival were observed for race, urban/rural residence, or census based poverty status.

## Discussion

Despite the powerful evidence that chemotherapy after surgery for Stage III colon cancer can improve survival, results of this study confirm previous studies that suggested that some groups of patients do not receive standard of care for this disease. Similar to other studies, age, marital status and comorbidity were major predictors of receipt of chemotherapy.^11^ For some individuals, adjuvant chemotherapy may not have been given due to contraindications from concurrent medical conditions. Our survival model suggested that adjuvant chemotherapy was protective among patients with and without comorbidities, and therefore seemed to suggest that, at least for some patients with higher comorbid illness burdens, adjuvant treatment can be protective. However, we did not have detailed information about severity of comorbid conditions within the categories used in our analysis to examine this further.

Geographic differences by state persisted after adjustment for other factors, and have not been studied as extensively. Interstate differences in receipt of treatment are likely a result of local practice patterns, lack of uniform acceptance of this treatment, and lack of access to treatment facilities. Regional differences in receipt of treatment also were seen in an earlier California study,^11^ as well as variation by hospital. In a survey of physicians, the most common reasons cited for not providing adjuvant chemotherapy for Stage III colon cancer were patient refusal, comorbid illness, and lack of clinical indication.^11^ Despite NIH recommendations, over 20% of physicians reported that adjuvant chemotherapy was not clinically indicated for their Stage III colon cancer patients.^11^ Interestingly, interstate differences in receipt of chemotherapy did not appear to correlate with interstate differences in survival, and it is likely that other factors are involved. This may be attributable in part to differences by state in the number of cases excluded from the survival analysis. Survival after diagnosis for patients in this study was influenced by age, comorbid illness, and receipt of chemotherapy.

Receipt of adjuvant treatment for patients in NPCR states appeared to be slightly, although not substantially, lower than for patients in areas covered by the National Cancer Institute’s Surveillance, Epidemiology and End Results (SEER) program. Results from SEER Patterns of Care studies reported that use of adjuvant chemotherapy for Stage III colon cancer increased between 1989 and 1990 when the NIH consensus conference was held, and has remained relatively stable since 1990.^12^ Receipt of chemotherapy in 1995 by patients with Stage III colon cancer in the SEER study was 90% for patients aged 55 or younger. This is slightly higher than the 86% of patients aged 50 or younger in the current study diagnosed in 1997. There is some evidence that patients residing in SEER areas are somewhat more affluent than other areas of the U.S,^13^ and they may have greater access to high quality cancer care. Receipt of treatment for older patients in the current study was comparable to that for similarly aged patients studied using linked SEER-Medicare data. Treatment rates for patients diagnosed with Stage III colon cancer between 1991 and 1996 and identified through the SEER program were 78% for those aged 65 to 69, and 74% for those aged 70–74, similar to the 75% for patients aged 65–74 in the current study diagnosed in 1997.^14^

Patients residing in neighborhoods with less education were less likely to have received adjuvant chemotherapy, although residence in poor neighborhoods did not have a statistically significant effect after adjustment for other factors. Patients residing in rural areas of the U.S. were not substantially less likely to have received treatment. Adjuvant chemotherapy is usually administered in outpatient treatment facilities and does not require the access to specialized treatment facilities required by adjuvant radiation treatment. Type of health insurance did not have a significant effect on receipt of treatment after adjustment for other factors, and did not influence survival. The majority of patients in the analysis were aged 65 or older and would have been covered by Medicare.

Our study was subject to several limitations. First, data were collected from seven states and are therefore not necessarily generalizable to all states. Moreover, data for this study were collected five years after the cancer diagnosis year, and therefore some patient records were not retrievable. Additionally, completeness of chemotherapy data may vary by registry. Our measures of socioeconomic status were derived from Census data and were therefore not individual-level data. Furthermore, we only collected comorbidities that had been coded on the face sheets of patients’ medical records. Therefore, comorbidities may have been underreported. Finally, we were unable to examine cause-specific mortality in addition to overall mortality.

The CDC-NPCR Patterns of Care study follows the National Cancer Policy Board recommendation that data systems such as the NPCR be used to conduct surveillance of cancer treatment in the United States. The study demonstrated the need for surveillance of cancer treatment across the U.S. by confirming gaps in treatment for some groups. This study also documented the substantial challenges to treatment surveillance using cancer registry treatment data. Analysis of quality of registry treatment data for patients in this study showed that only about 71% of chemotherapy received by colon cancer patients was captured by cancer registries through routine data collection.^6^ Use of registries for treatment surveillance will continue to require that treatment contained in the NPCR registries, as in SEER registries, be supplemented with data collection in physician offices and other treatment facilities.

## Figures and Tables

**Table 1.** Distribution of patients with stage II colon cancer by sex, sociodemographic and health factors in selected areas of the United States, 1997 (n = 973).

	**Total n (%)**	**Males n (%)**	**Females n (%)**
All cancers	973 (100)	461 (100)	512 (100)
Age at diagnosis (y)			
<50	113 (12)	61 (13)	52 (10)
50–64	259 (27)	132 (29)	127 (25)
65–74	267 (27)	132 (29)	135 (26)
≥75	334 (34)	136 (30)	198 (39)
Race/Ethnicity			
Hispanic	47 (5)	24 (5)	23 (4)
Non-Hispanic White	736 (76)	349 (76)	387 (76)
Non-Hispanic Black	164 (17)	73 (16)	91 (18)
Other/unknown	26 (3)	15 (3)	11 (2)
Marital status			
Married	558 (57)	326 (71)	232 (45)
Single	89 (9)	46 (10)	43 (8)
Other^a^	304 (31)	80 (17)	224 (44)
Unknown	22 (2)	9 (2)	13 (3)
Registry			
Colorado	157 (16)	85 (18)	72 (14)
Louisiana	197 (20)	93 (20)	104 (20)
Illinois	163 (17)	83 (18)	80 (16)
California	138 (14)	61 (13)	77 (15)
Rhode Island	80 (8)	28 (6)	52 (10)
South Carolina	104 (11)	39 (8)	65 (13)
New York	134 (14)	72 (16)	62 (12)
Health Insurance^b^			
Private	676 (69)	319 (69)	357 (70)
Public	198 (20)	92 (20)	106 (21)
None	33 (3)	19 (4)	14 (3)
Other/unknown	66 (7)	31 (7)	35 (7)
Residence^c^			
100% Urban	654 (67)	306 (66)	348 (68)
Urban-Rural Mix	254 (26)	117 (25)	137 (27)
100% Rural	63 (6)	37 (8)	26 (5)
Missing	2 (0.2)	1 (0.2)	1 (0.2)
Poverty^c^,^d^			
In Poverty	273 (28)	112 (24)	161 (31)
Not in Poverty	698 (72)	348 (75)	350 (68)
Missing	2 (0.2)	1 (0.2)	1 (0.2)
Education^c^,^e^			
Higher educational attainment	679 (70)	324 (70)	355 (69)
Lower educational attainment	292 (30)	136 (30)	156 (30)
Missing	2 (0.2)	1 (0.2)	1 (0.2)
Working class^c^,^f^			
Non-working class	465 (48)	218 (47)	247 (48)
Working class	506 (52)	242 (52)	264 (52)
Missing	2 (0.2)	1 (0.2)	1 (0.2)
Comorbidities^g^			
0	734 (75)	341 (74)	394 (77)
1	181 (19)	92 (20)	89 (17)
2+	58 (6)	29 (6)	29 (6)

a Other includes divorced, separated, and widowed.

b Private insurance includes those with private insurance or Medicare with supplement. Public insurance includes those with Medicare, Medicaid or welfare, or other federally funded health insurance.

c Data derived from the 2000 U.S. Census according to the census tract of the patient’s address.

d In poverty includes adults residing in census tracts where at least 20% of residents lived below the 2000 Federal Poverty Level.

e Higher educational attainment includes adults residing in areas where <25% of adults have less than a high school education. Lower educational attainment includes adults residing in areas where ≥25% of adults have less than a high school education.

f Non-working class includes adults residing in areas where <66% of adults are in a working class occupation. Working class includes adults residing in areas where ≥66% of adults are in a working class occupation.

g Determined by Charlson score.

**Table 2.** Proportion of patients with stage II colon cancer with known treatment status^a^ receiving adjuvant chemotherapy, by sex, sociodemographic and health factors in selected areas of the United States, 1997 (n = 915).

		**Number who received adjuvant chemotherapy**					
	**Total n**	**Males and Females n (%)**	**p**	**Males n (%)**	**p**	**Females n (%)**	**p**
All cancers	915^a^	614 (67)		297 (69)		317 (65)	
Age at diagnosis (y)			<0.0001		<0.0001		<0.0001
<50	111	95 (86)		52 (85)		43 (86)	
50–64	246	204 (83)		102 (82)		102 (84)	
65–74	248	187 (75)		87 (73)		100 (78)	
≥75	310	128 (41)		56 (44)		72 (39)	
Race/Ethnicity			0.94		0.46		0.54
Hispanic	43	28 (65)		16 (76)		12 (55)	
Non-Hispanic White	696	470 (68)		230 (70)		240 (65)	
Non-Hispanic Black	152	101 (66)		42 (62)		59 (70)	
Other/unknown	24	15 (63)		9 (64)		6 (60)	
Marital status			<0.0001		<0.0001		0.001
Married	530	395 (75)		229 (75)		166 (74)	
Single	86	56 (65)		31 (69)		25 (61)	
Formerly married^b^	282	153 (54)		34 (47)		119 (57)	
Unknown	17	10 (59)		3 (50)		7 (64)	
Registry			0.07		0.44		0.12
Colorado	151	109 (72)		62 (77)		47 (67)	
Louisiana	187	111 (59)		56 (62)		55 (57)	
Illinois	156	102 (65)		49 (64)		53 (67)	
California	130	82 (63)		41 (71)		41 (57)	
Rhode Island	76	55 (72)		19 (70)		36 (73)	
South Carolina	94	69 (73)		23 (70)		46 (75)	
New York	121	86 (71)		47 (73)		39 (68)	
Health Insurance^c^			0.001		0.02		0.04
Private	633	443 (70)		216 (72)		227 (68)	
Public	190	107 (56)		51 (59)		56 (54)	
None	31	26 (84)		15 (83)		11 (85)	
Other/unknown	61	38 (62)		15 (56)		23 (68)	
Residence^d^			0.20		0.42		0.42
100% Urban	614	409 (67)		192 (68)		217 (66)	
Urban-Rural mix	237	156 (66)		76 (70)		80 (63)	
100% Rural	62	48 (77)		29 (78)		19 (76)	
Poverty^d^,^e^			0.41		0.10		0.67
In Poverty	258	168 (65)		66 (63)		102 (67)	
Not in Poverty	655	445 (68)		231 (71)		214 (65)	
Education^d^,^f^			0.009		0.02		0.15
Higher educational attainment	639	446 (70)		219 (73)		227 (67)	
Lower educational attainment	274	167 (61)		78 (61)		89 (61)	
Working class^d^,^g^			0.97		0.36		0.43
Non-working class	436	293 (67)		147 (71)		146 (63)	
Working class	477	320 (67)		150 (67)		170 (67)	
Comorbidities^h^			<0.0001		0.001		0.002
0	689	488 (71)		233 (74)		255 (68)	
1	173	105 (61)		52 (60)		53 (62)	
2+	53	21 (40)		12 (44)		9 (35)	

a 58 individuals missing information about receipt of adjuvant chemotherapy, 2 individuals missing information about residence, poverty, education and working class.

b Formerly married includes divorced, separated, and widowed.

c Private insurance includes those with private insurance or Medicare with supplement. Public insurance includes those with Medicare, Medicaid or welfare, or other federally funded health insurance.

d Data derived from the 2000 U.S. Census according to the census tract of the patient’s address.

e In poverty includes adults residing in census tracts where at least 20% of residents lived below the 2000 Federal Poverty Level.

f Higher educational attainment includes adults residing in areas where <25% of adults have less than a high school education. Lower educational attainment includes adults residing in areas where ≥25% of adults have less than a high school education.

g Non-working class includes adults residing in areas where <66% of adults are in a working class occupation. Working class includes adults residing in areas where ≥66% of adults are in a working class occupation.

h Determined by Charlson score.

**Table 3.** Crude and adjusted odds ratios for receipt of adjuvant chemotherapy for Stage II colon cancer, by sociodemographic and health factors in selected areas of the United States, 1997.

	**Crude OR (95% CI) (n = 915^a^)**	**Model 1^b^ OR (95% CI) (n = 915^a^)**	**Model 2^b^ OR (95% CI) (n = 913^a^)**
Age at diagnosis (y)			
<50	1.94^j^ (1.06–3.54)	1.95^j^ (1.06–3.58)	1.68 (0.88–3.21)
50–64	1.58^j^ (1.02–2.46)	1.60^j^ (1.03–2.49)	1.48 (0.93–2.37)
65–74	1.00	1.00	1.00
≥75	0.23^j^ (0.16–0.334)	0.22^j^ (0.15–0.32)	0.24^j^ (0.16–0.35)
Sex			
Male	1.18 (0.90–1.56)	1.01 (0.74–1.37)	0.91 (0.65–1.27)
Female	1.00	1.00	1.00
Race/Ethnicity			
Hispanic	0.90 (0.47–1.71)	0.73 (0.36–1.47)	0.76 (0.36–1.61)
Non-Hispanic White	1.00	1.00	1.00
Non-Hispanic Black	0.95 (0.66–1.38)	0.73 (0.49–1.10)	1.06 (0.66–1.72)
Other/unknown	0.80 (0.35–1.86)	0.63 (0.25–1.57)	0.85 (0.32–2.23)
Marital status			
Married	1.00	1.00	1.00
Single	0.64 (0.39–1.04)	0.61 (0.36–1.05)	0.60 (0.34–1.04)
Formerly married^c^	0.41^j^ (0.30–0.55)	0.63^j^ (0.44–0.91)	0.59^j^ (0.41–0.86)
Unknown	0.49 (0.18–1.31)	0.56 (0.19–1.64)	0.48 (0.16–1.43)
Registry			
Colorado	1.78^j^ (1.12–2.82)	1.99^j^ (1.18–3.37)	1.92^j^ (1.10–3.36)
Louisiana	1.00	1.00	1.00
Illinois	1.29 (0.83–2.01)	1.33 (0.82–2.17)	1.24 (0.73–2.09)
California	1.17 (0.74–1.85)	1.19 (0.70–2.03)	1.41 (0.80–2.49)
Rhode Island	1.79^j^ (1.00–3.21)	2.20^j^ (1.15–4.21)	2.57^j^ (1.28–5.18)
South Carolina	1.89^j^ (1.10–3.25)	1.86^j^ (1.02–3.39)	1.60 (0.81–3.15)
New York	1.68^j^ (1.03–2.75)	1.88^j^ (1.09–3.26)	2.23^j^ (1.23–4.04)
Health Insurance^d^			
Private	1.00	1.00	1.00
Public	0.55^j^ (0.40–0.77)	0.71 (0.49–1.02)	0.73 (0.49–1.07)
None	2.23 (0.84–5.90)	0.98 (0.36–2.66)	1.05 (0.38–2.95)
Other/unknown	0.71 (0.41–1.22)	0.57 (0.31–1.05)	0.59 (0.32–1.11)
Residence^e^			
100% Urban	1.00	1.00	1.00
Urban-Rural mix	0.97 (0.70–1.33)	0.90 (0.63–1.27)	0.86 (0.59–1.26)
100% Rural	1.72 (0.93–3.19)	1.64 (0.84–3.22)	1.63 (0.79–3.35)
Poverty^e^,^f^			
In Poverty	0.88 (0.65–1.19)	1.05 (0.74–1.50)	1.25 (0.79–1.99)
Not in Poverty	1.00	1.00	1.00
Education^e^,^g^			
Higher educational attainment	1.00	1.00	1.00
Lower educational attainment	0.68^j^ (0.50–0.91)	0.74 (0.52–1.04)	0.61^j^ (0.38–0.99)
Working class^e^,^h^			
Non-working class	1.00	1.00	1.00
Working class	1.00 (0.75–1.31)	1.14 (0.84–1.56)	1.55^j^ (1.04–2.32)
Comorbidities^i^			
0	1.00	1.00	1.00
1	0.64^j^ (0.45–0.90)	0.81 (0.56–1.19)	0.79 (0.53–1.17)
2+	0.27^j^ (0.15–0.48)	0.42^j^ (0.22–0.79)	0.36^j^ (0.19–0.70)

a 58 individuals missing information about receipt of adjuvant chemotherapy. An additional 2 were missing information about urban residence, poverty, education and working class.

b Model 1 adjusted for age, sex, and race/ethnicity. Model 2 adjusted for all factors in the table.

c Formerly married includes divorced, separated, and widowed.

d Private insurance includes those with private insurance or Medicare with supplement. Public insurance includes those with Medicare, Medicaid or welfare, or other federally funded health insurance.

e Data derived from the 2000 U.S. Census according to the census tract of the patient’s address.

f In poverty includes adults residing in census tracts where at least 20% of residents lived below the 2000 Federal Poverty Level.

g Higher educational attainment includes adults residing in areas where <25% of adults have less than a high school education. Lower educational attainment includes adults residing in areas where ≥25% of adults have less than a high school education.

h Non-working class includes adults residing in areas where <66% of adults are in a working class occupation. Working class includes adults residing in areas where ≥66% of adults are in a working class occupation.

i Determined by Charlson score.

j p < 0.05

**Table 4.** Five-year survival and crude and adjusted hazard ratios for patients with stage II colon cancer by sex, sociodemographic and health factors in selected areas of the United States, 1997 (n = 883^a^).

	**5 yr survival (%)**	**Crude hazard Ratio (95% CI)**	**Adjusted^a^HR (95% CI) n = 881**
All cases	51.2		
Age at diagnosis (y)			
<50	61.8	0.73 (0.51–1.05)	
50–64	59.5	0.79 (0.60–1.04)	
65–74	53.1	1.00	
≥75	39.2	1.58^i^ (1.25–1.99)	
Sex			
Male	48.1	1.18 (0.98–1.42)	1.21 (0.99–1.47)
Female	53.9	1.00	1.00
Race/Ethnicity			
Hispanic	47.5	1.11 (0.71–1.72)	1.07 (0.67–1.71)
Non-Hispanic White	51.7	1.00	1.00
Non-Hispanic Black	49.6	1.04 (0.81–1.34)	0.90 (0.68–1.21)
Other/unknown	41.9	1.16 (0.67–2.02)	1.11 (0.62–2.00)
Marital status			
Married	53.8	1.00	1.00
Single	49.5	1.19 (0.85–1.65)	1.09 (0.77–1.56)
Formerly married^b^	45.0	1.40^i^ (1.14–1.71)	1.22 (0.97–1.52)
Unknown	64.2	0.66 (0.30–1.49)	0.67 (0.29–1.55)
Registry			
Colorado	46.6	0.98 (0.74–1.32)	1.30 (0.93–1.81)
Louisiana	45.0	1.00	1.00
Illinois	48.2	0.87 (0.64–1.17)	0.94 (0.68–1.31)
California	62.5	0.61^i^ (0.44–0.87)	0.62^i^ (0.42–0.90)
Rhode Island	62.6	0.59^i^ (0.39–0.89)	0.62^i^ (0.40–0.97)
South Carolina	46.9	0.85 (0.59–1.22)	1.01 (0.66–1.54)
New York	49.1	0.92 (0.68–1.26)	1.04 (0.74–1.47)
Health Insurance^c^			
Private	54.5	1.00	1.00
Public	42.4	1.44^i^ (1.16–1.80)	1.14 (0.90–1.44)
None	48.3	1.05 (0.63–1.77)	1.37 (0.79–2.38)
Other/unknown	46.1	1.23 (0.85–1.77)	1.31 (0.89–1.92)
Residence^d^			
100% Urban	51.7	1.00	1.00
Urban-Rural mix	52.0	1.04 (0.84–1.29)	1.00 (0.80–1.26)
100% Rural	41.4	1.22 (0.84–1.75)	1.15 (0.78–1.70)
Poverty^d^,^e^			
In Poverty	49.9	1.06 (0.86–1.30)	0.92 (0.70–1.22)
Not in Poverty	51.8	1.00	1.00
Education^d^,^f^			
Higher educational attainment	53.5	1.00	1.00
Lower educational attainment	45.7	1.26^i^ (1.04–1.54)	1.34^i^ (1.00–1.79)
Working class^d^,^g^			
Non-working class	53.5	1.00	1.00
Working class	49.0	1.15 (0.96–1.39)	0.96 (0.75–1.22)
Comorbidities^h^			
0	55.6	1.00	1.00^j^
1	40.0	1.58^i^ (1.26–1.99)	
Among those who received chemotherapy			1.08 (0.76–1.54)
Among those who did not receive chemotherapy			2.08 (1.50–2.87)
2+	26.0	2.26^i^ (1.62–3.17)	
Among those who received chemotherapy			1.62 (0.84–3.11)
Among those who did not receive chemotherapy			2.03 (1.32–3.12)
Adjuvant chemotherapy			
No	32.5	1.00	1.00^j^
Yes	60.8	0.41^i^ (0.34–0.50)	
Among those with no comorbidities			0.57 (0.44–0.73)
Among those with 1 comorbidity			0.30 (0.19–0.45)
Among those with 2+ comorbidities			0.45 (0.22–0.95)

a Adjusted for age and factors in the table. 90 individuals were missing information about whether or not they received chemotherapy or the time interval between diagnosis to receipt of chemotherapy. An additional 2 individuals were missing information about poverty, education, urban residence and working class. Therefore for those factors, n = 881.

b Formerly married includes divorced, separated, and widowed.

c Private insurance includes those with private insurance or Medicare with supplement. Public insurance includes those with Medicare, Medicaid or welfare, or other federally funded health insurance.

d Data derived from the 2000 U.S. Census according to the census tract of the patient’s address.

e In poverty includes adults residing in census tracts where at least 20% of residents lived below the 2000 Federal Poverty Level.

f Higher educational attainment includes adults residing in areas where <25% of adults have less than a high school education. Lower educational attainment includes adults residing in areas where ≥25% of adults have less than a high school education.

g Non-working class includes adults residing in areas where <66% of adults are in a working class occupation. Working class includes adults residing in areas where ≥66% of adults are in a working class occupation.

h Determined by Charlson score.

i p < 0.05

j interaction between comorbidity and adjuvant chemotherapy use significant, p < 0.05.

## References

[1] U.S. Cancer Statistics Working Group United States Cancer Statistics: 1999–2004 Incidence and Mortality Web-based Report Atlanta U.S. Department of Health and Human Services, Centers for Disease Control and Prevention and National Cancer Institute 2007

[2] NIH consensus conference Adjuvant therapy for patients with colon and rectal cancer JAMA 1990 264 1444 50 2202842

[3] Hodgson DC Fuchs CS Ayanian JZ Impact of patient and provider characteristics on the treatment and outcomes of colorectal cancer J Natl Cancer Inst 2001 93 501 15 11287444 10.1093/jnci/93.7.501

[4] Hewitt MaS JV Ensuring Quality Cancer Care Washington, DC National Academy Press 1999 25101447

[5] Hewitt MaS JV Enhancing data systems to improve the quality of cancer care Washington, DC National Academy Press 2000 25057727

[6] German RR Wike JM Wolf HJ Quality of Cancer Registry Data: Findings From CDC-NPCR’s Breast, Colon, and Prostate Cancer Data Quality and Patterns of Care Study Journal of Registry Management 2008 35 2 67 74 22096878

[7] McDavid K Schymura MJ Armstrong L Rationale and design of the National Program of Cancer Registries’ Breast, Colon, and Prostate Cancer Patterns of Care Study Cancer Causes Control 2004 15 1057 66 15801489 10.1007/s10552-004-1555-5

[8] SEER Program: Comparative staging guide for cancer, version 1.1 Bethesda National Institutes of Health, National Cancer Institute 1993

[9] Deyo RA Cherkin DC Ciol MA Adapting a clinical comorbidity index for use with ICD-9-CM administrative databases J Clin Epidemiol 1992 45 613 9 1607900 10.1016/0895-4356(92)90133-8

[10] Krieger N Overcoming the absence of socioeconomic data in medical records: validation and application of a census-based methodology Am J Public Health 1992 82 703 10 1566949 10.2105/ajph.82.5.703 PMC1694121

[11] Ayanian JZ Zaslavsky AM Fuchs CS Use of adjuvant chemotherapy and radiation therapy for colorectal cancer in a population-based cohort J Clin Oncol 2003 21 1293 300 12663717 10.1200/JCO.2003.06.178

[12] Potosky AL Harlan LC Kaplan RS Age, sex, and racial differences in the use of standard adjuvant therapy for colorectal cancer J Clin Oncol 2002 20 1192 202 11870160 10.1200/JCO.2002.20.5.1192

[13] Warren JL Klabunde CN Schrag D Overview of the SEER-Medicare data: content, research applications, and generalizability to the United States elderly population Med Care 2002 40 IV-3 18 10.1097/01.MLR.0000020942.47004.03 12187163

[14] Schrag D Cramer LD Bach PB Age and adjuvant chemotherapy use after surgery for stage III colon cancer J Natl Cancer Inst 2001 93 850 7 11390534 10.1093/jnci/93.11.850

